# “Our focus is on illness and loneliness”: Volunteer work engagement, compassion satisfaction, compassion fatigue, self‐care and motivations to volunteer

**DOI:** 10.1111/hsc.13934

**Published:** 2022-07-27

**Authors:** Ana J. Cañas‐Lerma, José Francisco Campos‐Vidal, Sebastià Verger

**Affiliations:** ^1^ Department of Philosophy and Social Work Universitat de les Illes Balears Palma Spain; ^2^ Department of Applied Pedagogy and Educational Psychology Universitat de les Illes Balears Palma Spain

**Keywords:** volunteer compassion fatigue, volunteer compassion satisfaction, volunteer functions inventory, volunteer self‐care, volunteer work engagement

## Abstract

They are participating as a volunteer implies active personal positioning accompanying others. Evidence supports that experiences of those who experience an illness, who are hospitalised or feel lonely, impact the volunteers: positive emotions like engagement and Compassion Satisfaction (CS) or, the reverse, Compassion Fatigue (CF). Motivations help us understand why volunteers spend their time on these activities. And self‐care practices will be a challenge to counteract the exhausting emotions of volunteering. This research presents a mixed, exploratory and sequential design study on the island of Majorca (Spain). The first phase (*n* = 216) was quantitative, gathering data from November 2018 to April 2019. Then, the second phase (two focus groups) started with qualitative data collection (July 2019). Firstly, the study determines CS and work engagement levels and examines the relationship between self‐care, CF and motivations. Secondly, the study finds out how they recognise their positive and negative emotions, their relationship with self‐care and what motivates them to be volunteers. The results show that the volunteers report highly positive feelings associated with their volunteering (CS and engagement) and are backed up by a good level of personal Self‐Care. The Understanding and Enhancement motivational functions generate even more positive feelings for the volunteers themselves, who attach a positive value to their experience of caring for others. Despite the positive results collected, we must not ignore the phenomenon of CF in relational volunteering and pain support because it occurs. After all, that could lead to abandonment by volunteers.

**What is known about this topic**

- Compassion Satisfaction and Compassion Fatigue are developed significantly in professional areas like nursing and social work, but not in volunteering.
- Self‐care in volunteering is not widely investigated, and it is necessary to continue expanding knowledge.
- Social workers and volunteer organisations consider the motivations of volunteers to promote volunteering.

**What this paper adds**

- High levels of Compassion Satisfaction and engagement generate continuity of the person volunteering with the same organisation.
- Memorable pain‐related experiences have given positive value to their volunteer experience.
- The results obtained are of interest to increase the permanence of volunteers from the social‐health field.

## 1 INTRODUCTION

Participating as a volunteer in the community individually or as part of a group implies active personal positioning to respond to situations of scarcity or need that we recognise in our surroundings. This paper considers these needs to be satisfied, not only from private or public organisations but also from citizens. Therefore, volunteering is not only a leisure time option but rather a prosocial, planned, not sporadic behaviour carried out within the framework of an organisation and aimed at the care of others (Penner, 2002). Thus, volunteering in health and social care is impossible to carry out individually. It bases on establishing a helping relationship with the other who experience loneliness or is affected, the person or a family member, by an illness. It would represent volunteering recognised within the primary duality as a form of collective organisation, presented by Hustinx (2010); that is, a person decides to volunteer to change the situation they consider unfair from their mobilising interest.

Pre‐COVID‐19 time, in Spain, 6.7% of people over 14 years of age participated as volunteers in 2019 – more than half of these were women involved mainly in care‐related volunteering such as the field of health and social care (Plataforma del Voluntariado de España, 2019 [Spanish Volunteering Platform]; Plataforma de ONG de Acción Social, 2020 [Social Accion NGO's Platform]). These are volunteers who devote their time to accompanying people in hospital settings (Pérez et al., 2018), in palliative care (Arantzamendi & Centeno, 2017), providing grief support (Becerra et al., 2017; Carneiro et al., 2009) or devoting time and care to someone affected by dementia (Van Der Ploeg et al., 2012).

Thus, according to the categorization of prosocial behaviours offered by Aydinli et al. (2013), we recognise in this volunteering a behaviour that helps others. It is from the establishment of this helping relationship, that the type of “relational volunteering” (Cañas‐Lerma, 2020) analysed in this research is built. It is a type of volunteering linked to a high emotional load. Establishing a helping relationship is essential because it is volunteer work focused on caring for the person who experiences suffering and aimed at alleviating their discomfort. Therefore, our empathy plays a fundamental role in offering this therapeutic relationship to the other. It is empathy that brings us closer to the experience of the other, to understand “as if” it was happening to us and offering ourselves, from our role as volunteers, to care for the other from a compassionate and considerate perspective. Yet it is at this point where we emphasise the social‐health volunteers. By offering themselves as a care resource, they become exposed to the inference in them of positive feelings and more complex and exhausting feelings deriving from the pain, discomfort or injustice experienced by others. Being permeable beings that offer themselves as a care resource will make them infer positive motivations and emotions ‐Compassion Satisfaction and volunteer work engagement‐ and other more exhausting and complex ones ‐Compassion Fatigue (Hidalgo‐Andrade & Martínez‐Rodríguez, 2019; Meneghini et al., 2018; Montross‐Thomas et al., 2016). For this reason, it is vital for the wellbeing of the volunteers that they become aware of themselves and participate in or promote self‐care strategies to elevate or maintain a good level of positive feelings. In this sense, this study connects the first information approach of this type, including self‐care practices in volunteering.

### 1.1 Compassion satisfaction

Establishing a caring relationship based on empathy can generate positive emotions for us. Feeling connected to others is an essential component of compassion, and participating in volunteering, which produces feelings of commitment and interconnection with others, arouses satisfaction in us (Snyderman & Gyatso, 2019). For Stamm (2010), Compassion Satisfaction is one of these positive emotions. She defines it as the personal reward generated by the knowledge that we are offering ourselves as a therapeutic resource to others. West et al. (2018) point out that it reflects our resilience and ability to give a meaning of enrichment and personal growth to those stressful experiences and/or relationships. Some factors that can help increase Compassion Satisfaction in support's volunteers in pain, illness or loneliness are the use of personal rituals (Montross‐Thomas et al., 2016) or self‐perception of having social support (Di Marco et al., 2020). To participate in a flexible environment, accepting death as part of life, and knowing how to maintain an adequate personal and emotional distance (Guirguis‐Younger & Grafanaki, 2008) are others strategies.

### 1.2 Compassion fatigue

The emotional cost involved in caring for others from a relationship based on empathy is what Figley (1995a) recognises as Compassion Fatigue. It appears when the person is emotionally exposed to the pain and discomfort of the other and does not have personal or organisational support to cope with this unpleasant situation (Figley, 1995b). Signs that indicate a high level of Compassion Fatigue can arise quickly. It may well be related to one harrowing experience in particular or long‐term exposure to different understandings of the pain of others (Alkema et al., 2008). Some of these signs are apathy, annoying thoughts or mental images related to the other's painful experience, questioning of spiritual beliefs, isolation and insomnia, among many others (Absolon & Krueger, 2009; Figley, 1995b; Kreutzer & Jager, 2011; Moreno‐Jiménez et al., 2004). In addition to the factors Figley (1995b) recognises as generators of high Compassion Fatigue the absence of self‐care behaviours, unresolved personal trauma, high tension derived from an inability to handle the work, and lack of feelings of satisfaction concerning the work carried out. In health and social care volunteering, there are other factors non‐protective to experience Compassion Fatigue. These factors are lack of a volunteer code of ethics (Avieli et al., 2016), having anxiety or depression (Jo et al., 2020), and dealing with the death of a user, among others (Pöyhiä et al., 2019).

### 1.3 Engagement

Participating in a volunteer means doing it under the umbrella of an organisation. In this sense, strengthening and emotional management are significant, from the organisations themselves (Salanova et al., 2016), including those that manage volunteer programs. From this approach, engagement is the positive feeling related to the work carried out, not specifically with a particular activity or task, but in general terms (Schaufeli et al., 2002). Despite being a term initially linked to work environments, Vecina et al. (2012) analysed it with a sample of volunteers, obtaining significant results. Engagement is made up of three constructs. For Schaufeli et al. (2002), vigour is the ability, despite difficulties, to be resilient, to strive and persevere in whatever is being done in the organisational setting; dedication is an excellent feeling of significance with the role played within the organisation and the pride this generates, in this case being a volunteer. Finally, absorption is the ability to control a situation, the enjoyment of concentrating on what is being done and the feeling that time passes fast while performing the role.

### 1.4 Motivational factors for volunteering

In addition to the pleasant feelings of Compassion Satisfaction, and Engagement, other individual elements intercede in the causes whereby a person starts or carries on health and social care volunteering (Selli et al., 2008). Recent studies reported a positive association between older people's volunteering, cognitive functioning (Guiney et al., 2021; Han et al., 2020), physical health (Pettigrew et al., 2020) and acquisition of skills or increased social connectedness (Jongenelis et al., 2020; Russell et al., 2019; Wilding et al., 2021). Authors are according with Haski‐Leventhal's (2009) theoretical proposal based on altruistic motivations or civic concerns. Likewise, this study shares the approach of Clary and Snyder (1991). After a bibliographic review, they stated that greater satisfaction and effectiveness in volunteering occurred in those cases in which the volunteer work established a psychological function for the volunteers themselves. The functional motivation theory (Clary et al., 1992) adopts a functional approach along these lines. It develops a model of six motivational factors for volunteering: (1) Values: volunteering is an opportunity to manifest values related to altruistic or humanitarian concerns for others. (2) Understanding: it is an opportunity for new learning experiences and practising one's own knowledge and skills. (3) Social: an opportunity to be with friends, to participate in an activity seen as positive by people important to me. (4) Career: a chance to promote benefits for one's professional career. (5) Protective: volunteering can be an opportunity to protect the ego. It can reduce the guilt of being more fortunate than others or address personal issues. (6) Enhancement: an opportunity to enhance good morale and maintain or improve positive affect. Chacón et al. (2017) offer a systematic review that collects studies about this motivational approach in health and social volunteers.

Different studies relate to motivation and its positive relationship with satisfaction and retaining volunteers (Faletehan et al., 2020; Meneghini et al., 2018; Mullan et al., 2021). In this sense, Ferreira et al. (2012, 2015), identified four different motivation categories with hospital volunteers, and their positive relationship with satisfaction and intention to stay: development and learning, altruism, protection and career recognition.

### 1.5 Self‐care is a strategy to appease negative feelings and maintain positive ones

The concept of self‐care is deeply rooted in the professional world but not in the same way in volunteering. There is a false myth about self‐care, stating that it is a selfish practice, but nothing could be further from the truth (Miller et al., 2018). It is about caring for oneself. This self‐care may be physical (activity and physical care in daily life), internal (taking care of one's inner world) and social (attending to the optimal care of our social and organisational relationships (Galiana et al., 2015)).

Relational volunteering provides support in a hospital or residential setting. It brings the volunteer close to significant personal experiences where affection, tenderness or joy appear and emotional or physical pain, frustration or illness. Therefore, this exposure to the feelings and realities of others requires self‐care guidelines to mitigate the negative impact on volunteers of the negative experiences that others share with them, either directly or indirectly. Examples of these practices include participation in group sessions as a space for openness where the feelings arising through volunteering can be shared (Haski‐Leventhal & Cnaan, 2009; Moreno‐Jiménez et al., 2013), mindfulness (Pintado & Giannini, 2016), therapeutic application of humour (Hatzipapas et al., 2017), and reflective writing (Vincett, 2018).

### 1.6 Purpose of the study

In the quantitative part of the study, the first objective was to determine the levels of compassion satisfaction, volunteer work engagement, self‐care, compassion fatigue and motivations in health and social care volunteers and examine the relationship between them. In the qualitative part, the second objective was to find out how participants recognise and process their positive and negative emotions (Compassion Satisfaction ‐CS, engagement and Compassion Fatigue ‐CF), their self‐care relationship and what motivates them to be volunteers in the sphere of illness and loneliness.

## 2 METHOD

### 2.1 Procedure and recruitment

This research has a mixed, exploratory and sequential design. The mixed‐methods design was chosen to highlight not only quantitative data but also participants' voice. Thus, quantitative data allow statistical procedures that contribute to offer guidelines in the population studied, and the qualitative approach allows the access to deepen in descriptions of the phenomena studied. The first phase was quantitative (Q‐1), gathering data from November 2018 to April 2019. The second was qualitative (Q‐2), gathering data in July 2019. The first author contacted with organisations (*n* = 17) offering volunteer programs for supporting people in palliative care, older adults with multiple pathologies with good cognitive ability or in a situation of unwanted loneliness, or persons in drug‐abuse treatment program. Volunteers visit them at home, in hospitals or older adults' homes on the island of Majorca (Spain). Sixteen organisations agreed to send our invitation to participate in the study out to their volunteers. E‐mails were sent to the volunteer coordinators explaining the purpose of the study and requesting their cooperation in the volunteer recruitment process. The coordinators of the volunteers organised the meetings as the researchers could not access their contact information in the Q‐1 phase. The participants in this phase had identified through snowball sampling and word‐of‐mouth sampling. According to the levels of CS and CF, the research team organised the inclusion criteria for the focus group participants' in the Q‐2 phase. At this point, the intention was to gain insight into the experience and meanings of support volunteers' expertise and their description of their CF emotions, recognising CS, engagement, motivations and self‐care practices in their volunteering.

### 2.2 Sample

Participants' characteristics are presented in Table 1.

**TABLE 1:** Participants' characteristics

		Participants quantitative part (Q‐1) *N* = 216			Participants qualitative part (Q‐2) *N* = 13		
		*n* (%)	Mean (SD)	Range	*n* (%)	Mean (SD)	Range
Gender	Female	170 (78.7)			9 (69.23)		
	Male	46 (21.3)			4 (30.77)		
Age		212 (98.1)	58.33 (16.868)	16–90	13 (100)	55 (17.776)	19–71
Did not answer		4 (1.9)			0 (0)		
Educational level	No studies	7 (3.2)			0 (0)		
	Primary school	54 (25)			2 (15.38)		
	Secondary school	17 (7.9)			3 (23.08)		
	Senior college	56 (25.9)			4 (30.77)		
	University degree	82 (38)			4 (30.77)		
Work	Retired	110 (50.9)			6 (46.15)		
	Paid work	65 (30.1)			6 (46.15)		
	Student	16 (7.4)			1 (7.69)		
	Unemployed	23 (10.6)			0 (0)		
Did not answer		2 (0.9)			0 (0)		
Length of experience volunteering months		215 (99.5)	81.96 (86.300)	5–480	13 (100)	113 (102.264)	8–360
Did not answer		1 (0.5)			0 (0)		
Days per week volunteering		212 (98.1)	1.4 (0.894)	1–6	13 (100)	1.3 (0.480)	1–2
Did not answer		4 (1.9)			0 (0)		

Abbreviation: SD, standard deviation.

#### 2.2.1 Participants quantitative part (Q‐1)

The criteria for being included in the Q‐1 phase were having at least 5 months' experience as a volunteer in one of the 16 organisations accessed to participate in the study, being active and recognising doing volunteering by establishing a supportive relationship. The number of participants who completed the survey was 216, aged between 16 and 90 years (M = 58.33, SD = 16.868), most of whom were female. Most of the participants were retired (*n* = 110) or paid workers (*n* = 65). No compensation was earned for participating. Their experience as a volunteer in this field varied from 5 months to 40 years, with 33% serving between 1 and 3 years. The number of days and hours per week participants reported working as volunteers ranged from 1 to 6 days and 1 to 12 h (M = 3.03, SD = 1.647). A total of 191 participant indicated their interest in continuing in qualitative part sharing their personal number phone. Participants' characteristics are presented in Table 1.

#### 2.2.2 Participants qualitative part (Q‐2)

Participants in the Q‐2 phase (*n* = 191) were selected according to their levels of CS and CF, using a random sampling procedure with Excel software. The final sample consisted of 13 participants divided into two focus groups. The selection of participants was from an intentional stratified sampling to find out and describe in detail the characteristics, either similar or different, which occur in the subgroups (Teddlie & Yu, 2007). The inclusion criteria for the first focus group (four women and three men, ranging in age from 24 to 71 years) were as follows: (1) In the Q‐1 phase, have indicated their interest in participating in the Q‐2 and providing their telephone number; (2) average CF; (3) high CS. The inclusion criteria for the second focus group (five women and one man, ranging in age from 19 to 70 years) were: (1) In the Q‐1 phase, have indicated their interest in participating in the Q‐2 and providing their telephone number; (2) low CF; (3) high CS. Participants were retired (*n* = 6) or paid workers (*n* = 6).

### 2.3 Instruments

#### 2.3.1 Quantitative part (Q‐1)

The sociodemographic form includes gender, age, level of education, work situation and volunteer‐related variables (length of volunteer service in months and total days).

Compassion satisfaction and Compassion fatigue: in this study, two of the three 10‐item subscales of the Spanish version of the Professional quality of life scale (ProQOL‐ Mendoza et al., 2014; Stamm, 2010) were used. The ProQOL is a common measure of the positive and negative effects of working with people who have experienced traumatic experiences (Stamm, 2010). With the permission of ProQOL.org, we exchanged “helper” or “help” for “volunteer” or “volunteering”. Responses are based on a five‐point scale, with anchor points ranging from 1 (never) to 5 (very often). Compassion satisfaction (CS) and compassion fatigue (CF) subscales were used (CS = 0.84, and CF = 0.68). As in Gonzalez‐Mendez et al. (2020), the burnout (BO) subscale revealed low internal consistency and, as such, was ruled out of this study (BO = 0.57). The following questions are representative items used from the CS scale (M = 43.19, SD = 5.088): “I like my work as a volunteer helping others”, “I believe I can make a difference through my volunteering”, and the CF scale (M = 18.99, SD = 4.510) “Because of my volunteering, I have felt”on edge“about various things”, “As a result of my volunteering, I have intrusive, frightening thoughts”.

Engagement with their volunteer work: the Spanish version of the Utrecht Work Engagement Scale 9‐item questionnaire was used (UWES‐ Schaufeli et al., 2002; Schaufeli & Bakker, 2004).

Work engagement (M = 5.1517, SD = 0.76562) is a construct composed of three dimensions: vigour, dedication and absorption. It is selected because although it is a construct that initially arises for the study of paid work people, volunteering management has many similarities with positivist organisational behaviours (Vecina et al., 2012). Responses are based on a three 3‐item subscale, with anchor points ranging from 0 (never) to 6 (always). In this study, the following Cronbach's alpha values were obtained for total engagement (α = 0.85), vigour (α = 0.76), dedication (α = 0.69) and absorption (α = 0.70). The following questions are representative items used from the three subscales. The author's permission was acquired in order to include “volunteer work” in questions to the dedication scale (M = 5.3580, SD = 0.78250) (e.g., “My volunteer work inspires me”), and absorption scale (M = 5.0941, SD = 0.98142) (e.g., “I am immersed in my volunteer work”). For the vigour scale (M = 5.0031, SD = 0.91668), permission was obtained to include the word “volunteering” (e.g., “When I get up in the morning, I feel like going to my volunteering”).

Motives for volunteering: the Spanish version of the Volunteer Functions Inventory (VFI‐ Clary et al., 1998; Dávila & Chacón, 2005) was used. Even though for Clary et al. (1998), the VFI has had its six‐factor structure corroborated, in our sample, Cronbach's alphas for the VFI subscales were only acceptable in five, ranging from 0.68 to 0.9, excluding subscale values (α = 0.57). The VFI consists of six 5‐item subscales, and responses are based on a seven‐point scale ranging from 1 (not at all important) to 7 (extremely important), rating the importance of each reason for volunteering. In this study, five total scores were computed for each participant by averaging items in each of the motive subscales: understanding function (M = 5.65, SD = 1.071) (e.g., “Volunteering allows me to gain a new perspective on things”); enhancement function (M = 4.58, SD = 1.502) (e.g., “Volunteering increases my self‐esteem”); social function (M = 4.19, SD = 1.520) (e.g., “My friends volunteer”); protective function (M = 3.13, SD = 1.310) (e.g., “By volunteering, I feel less lonely”) and career function (M = 2.01, SD = 1.477) (e.g., “Volunteering will help me succeed in my chosen profession”).

Self‐care: in this study, we adapted, with the author's permission, the Professional Self‐Care Scale (PSCP‐ Galiana et al., 2015) to our sample: volunteer people who work with ill/lonely people (M = 3.9923, SD = 0.69616). The word “volunteering” was used instead of “work.” And in the 9th item, “clinical situation” was changed for “emotional situation related with your volunteering.” A Cronbach alpha value of 0.737 was obtained. Responses are based on a nine‐item 5‐point Likert scale (from 1, “totally disagree” to 5, “totally agree”). Examples of the questions are “I do exercise regularly,” “I believe that my relations outside volunteering are satisfactory,” “My self‐care includes getting actively involved in spiritual practice, meditation, oration….”

#### 2.3.2 Qualitative part (Q‐2)

Focus groups: the two groups were facilitated using a question guide to prompt discussion. We adapted, by way of stimulator material (Barbour, 2013), the collective narrative practice “The tree of life” (Denborough, 2008). Our adaptation, “The Volunteering Tree”, in its first part, promoted reflection on and self‐knowledge of the individual volunteering experience of each participant. In the second part, we created “The Volunteering Forest” to encourage group responses (e.g., “Which positive and negative emotions does your volunteering generate for you?”), and finally, we explored what happened when difficult situations (“the storms”) impacted their volunteer experiences (e.g., “How do you recognize your affliction, your emotional fatigue, in your volunteering?; How protected are you from these situations?”).

### 2.4 Data collection and analysis

All the statistical data were analysed using IBM SPSS software version 25. The Kolmogorov–Smirnov test demonstrated the absence of normality in data distribution, so nonparametric statistics were used. Univariate and bivariate descriptive data analysis, correlations and contrast tests such as Mann–Whitney U, Kruskal–Wallis and Spearman's Rho were performed. Missing data were handled by using the “Exclude cases listwise” option in SPSS.

The two focus groups were digitally recorded, transcribed verbatim and coded using the constant comparative method: data reduction, unitizing, open coding and axial coding (Glaser & Strauss, 1967). To provide strong evidence for interpreting the data, investigator triangulation was used to analyse transcripts and develop emergent and adapted aprioristic themes independently. Data were analysed using Atlas.ti software version 7.5.4.

### 2.5 Ethical aspects

Due permission was obtained from the University of the Balearic Islands' Research Ethics Committee (Ref. 94CER18). All subjects in the sample voluntarily signed informed consent forms, having been informed of the purpose of the study and, in both phases, data confidentiality and participant anonymity was always ensured, following Spanish Organic Law 3/2018 of December 5 for the protection of personal data and the guarantee of digital rights, and respecting the Declaration of Helsinki. Finally, if the participant did not check the box to participate in the second phase and provide their telephone number, they were not allowed access to the focus groups (Q‐2).

## 3 RESULTS

Participants reported high compassion satisfaction (M = 43.19, SD = 5.08), low compassion fatigue (M = 18.99, SD = 4.51) and frequently looking after their self‐care (M = 4.03, SD = 0.79). Only 32.4% presented average compassion satisfaction level, and 21.8% compassion fatigue level. The results also show that volunteers are engaged with their participation (M = 5.15, SD = 0.76) and showed high levels of Vigour (M = 5, SD = 0.91), Dedication (M = 5.35, SD = 0.78) and Absorption (M = 5.09, SD = 0.98). Finally, for this sample, the following mean scores were found on reported importance for each motivation scale: Understanding (M = 5.65, SD = 1.07), Enhancement (M = 4.57, SD = 1.50), Social (M = 4.18, SD = 1.52), Protective (M = 3.12, SD = 1.30) and Career (M = 2.00, SD = 1.47).

Correlations between variables are shown in Table 2. Compassion Satisfaction (CS) is related positively to Engagement, Vigour, Dedication, Absorption and Understanding, Enhancement, Social and Protective volunteer functions. Compassion Fatigue (CF) also correlated positively with Enhancement, Social and Protective functions, while correlating negatively with Self‐Care, Vigour and Dedication. Engagement correlated very highly and positively with its sub‐scales and with age, Understanding, Enhancement, Social and Protective functions. Further, Vigour is positively related to age, Dedication, Absorption, Understanding, Enhancement, Social and Protective functions. Dedication positively correlated with Absorption, Understanding, Enhancement, Social and Protective. Last, Absorption is related positively to age, length of experience as a volunteer, and all functions of volunteer motivations apart from Career. Finally, volunteer motivation functions are also positively correlated with each other.

**TABLE 2:** Spearman's correlations between study variables Q‐I part

	1	2	3	4	5	6	7	8	9	10	11	12	13	14
1	‐													
2	0.468**	‐												
3	0.099	0.031	‐											
4	0.013	0.045	0.081	‐										
5	0.054	0.027	0.060	−0.095	‐									
6	0.085	0.085	−0.118	−0.031	−0.211**	‐								
7	0.218**	0.129	0.089	0.653**	−0.085	0.026	‐							
8	0.169*	0.084	0.108	0.527**	−0.169*	0.079	0.853**	‐						
9	0.077	0.076	0.063	0.681**	−0.152*	−0.033	0.815**	0.614**	‐					
10	0.273**	0.141*	0.099	0.496**	0.041	0.015	0.829**	0.557**	0.542**	‐				
11	−0.085	−0.035	0.147*	0.404**	0.006	0.073	0.394**	0.311**	0.421**	0.298**	‐			
12	0.134	−0.007	0.147*	0.414**	0.142*	−0.079	0.450**	0.371**	0.413**	0.359**	0.521**	‐		
13	0.087	0.179**	0.058	0.150*	0.142*	0.054	0.222**	0.187**	0.183**	0.167*	0.358**	0.478**	‐	
14	0.108	−0.002	0.193**	0.188**	0.214**	−0.060	0.291**	0.246**	0.280**	0.211**	0.420**	0.620**	0.440**	‐
15	−0.470**	−261**	0.087	0.087	0.104	−0.110	−0.012	−0.044	0.079	−0.046	0.250**	0.311**	0.189**	0.319**

*Notes*: 1 = age, 2 = length of experience as a volunteer/months, 3 = days per week as a volunteer, 4 = CS, 5 = CF, 6 = self‐care, 7 = engagement, 8 = vigour, 9 = dedication, 10 = absorption, 11 = understanding, 12 = enhancement, 13 = social, 14 = protective, 15 = career.

* *p* < 0.05

** *p* < 0.01.

Table 3 presents the distribution of mean scores for CS, CF, Self‐Care, Engagement and each UWES‐9 dimension, regarding the values of the gender and education level variables. Respecting the bivariate analysis, no significant differences were found in the mean questionnaire score for gender and education variables and CS and CF, whereas significant differences were encountered between gender and Self‐Care (U Mann–Whitney *p* < 0.01), and Engagement (U Mann–Whitney *p* < 0.05). Finally, education level was significant with Engagement (H Kruskal–Wallis *p* < 0.01), Dedication (H Kruskal–Wallis *p* < 0.05), and Absorption (H Kruskal–Wallis *p* < 0.01). In contrast, no significant differences were found in the mean questionnaire score for the labour situation variables and CS (H Kruskal–Wallis *p* = 0.420), CF (H Kruskal–Wallis *p* = 0.562), Self‐Care (H Kruskal–Wallis *p* = 0.561), Engagement (H Kruskal–Wallis p = 0.212), Vigour (H Kruskal–Wallis *p* = 0.249), Dedication (H Kruskal–Wallis *p* = 0.411) or Absorption (H Kruskal–Wallis *p* = 0.539).

**TABLE 3:** Distribution of mean scores by compassion satisfaction, compassion fatigue, self‐care and UWES‐9 dimension, and statistical significance with socio‐demographic variables

Variable	*n*	CS		CF		Self‐care		Engagement		Vigour		Dedication		Absorption	
		M (SD)	*p*‐value	M (SD)	*p*‐value	M (SD)	*p*‐value	M (SD)	*p*‐value	M (SD)	*p*‐value	M (SD)	*p*‐value	M (SD)	*p*‐value
Gender			0.973^d^		0.978^d^		**0.008** ^d^**		**0.052** ^d^*		0.028^d^		0.572^d^		0.095^d^
Male	46	43.28 (4.810)		18.91 (4.540)		3.76 (0.766)		5.00 (0.737)		4.731 (1.025)		5.347 (0.706)		4.935 (0.995)	
Female	170	43.16 (5.174)		19.01 (4.515)		4.11 (0.792)		5.19 (0.770)		5.076 (0.874)		5.360 (0.804)		5.137 (0.976)	
Education level			0.525^B^		0.783^B^		0.336^B^		**0.002** ^**B**^ **		0.068^B^		**0.059** ^B^*		**0.001** ^**B**^ **
None	7	44.86 (4.525)		19.43 (5.192)		3.57 (1.134)		5.651 (0.437)		5.287 (0.889)		5.808 (0.326)		5.857 (0.377)	
Primary	54	43.52 (4.596)		19.48 (4.390)		3.89 (0.861)		5.527 (0.769)		5.074 (0.981)		5.450 (0.682)		5.247 (1.038)	
Second.	17	44.82 (3.893)		18.88 (4.076)		3.94 (0.966)		5.331 (0.600)		5.254 (0.759)		5.510 (0.602)		5.234 (0.753)	
Senior college	56	42.79 (5.386)		19.14 (4.657)		4.07 (0.783)		5.246 (0.766)		5.082 (0.888)		5.495 (0.758)		5.202 (0.887)	
Univer.	82	42.77 (5.433)		18.54 (4.579)		4.16 (0.675)		4.937 (0.772)		4.825 (0.912)		5.162 (0.881)		4.825 (1.021)	

*Notes*: Second: Secondary, Univer: University, CS = Compassion Satisfaction, CF = Compassion Fatigue; ^d^: *U* Mann–Whitney; ^B^: H Kruskal–Wallis. Significant differences: *p < 0.05. **p < 0.01 are indicated in bold.

Sociodemographic variables of gender, education level and labour situation are shown in Table 4 for the distribution of mean scores for volunteer functions. Significant differences were found between education level and Enhancement (H Kruskal–Wallis *p* < 0.01), Social (H Kruskal–Wallis *p* < 0.01) and Protective (H Kruskal–Wallis *p* < 0.01) volunteer functions. Notably, the Career function revealed significant differences with the labour situation variable.

**TABLE 4:** Distribution of mean scores by volunteer functions and statistical significance

Variable	*n*	Understanding		Enhancement		Social		Protective		Career	
		M (SD)	*p*‐value	M (SD)	*p*‐value	M (SD)	*p*‐value	M (SD)	*p*‐value	M (SD)	*p*‐value
Gender			0.574^d^		0.881^d^		0.739^d^		0.864^d^		0.246^d^
Male	46	5.756 (0.983)		4.539 (1.440)		4.265 (1.456)		3.113 (1.255)		2.078 (1.369)	
Female	170	5.624 (1.095)		4.588 (1.522)		4.165 (1.540)		3.131 (1.327)		1.988 (1.507)	
Education level			0.788^B^		**0.017** ^B^**		**0.022** ^B^**		**0.006** ^B^**		0.301^B^
None	7	5.857 (1.231)		5.885 (1.727)		5.600 (1.070)		4.714 (1.459)		2.542 (2.371)	
Primary	54	5.551 (1.127)		4.822 (1.568)		4.255 (1.439)		3.425 (1.283)		1.722 (1.125)	
Secondary	17	5.882 (0.961)		4.917 (1.442)		4.823 (1.506)		2.870 (1.264)		2.058 (1.295)	
Senior	56	5.671 (1.012)		4.642 (1.281)		4.053 (1.374)		3.067 (1.330)		1.975 (1.534)	
University	82	5.641 (1.095)		4.190 (1.505)		3.980 (1.627)		2.890 (1.204)		2.161 (1.583)	
Labour situation			0.299^B^		0.064^B^		0.205^B^		0.864^B^		**0.053** ^B^**
Retired	110	5.721 (0.967)		4.538 (1.456)		4.269 (1.494)		3.070 (1.242)		1.952 (1.554)	
Paid work	65	5.590 (1.142)		4.695 (1.576)		4.163 (1.530)		3.230 (1.350)		1.876 (1.286)	
Student	16	6.025 (0.946)		5.200 (1.531)		4.625) (1.715)		3.150 (1.656)		3.062 (1.804)	
Unemployed	23	5.278 (1.369)		4.026 (1.408)		3.669 (1.436)		3.104 (1.237)		1.965 (1.170)	

*Notes*: ^d^: *U* Mann–Whitney; ^B^: H Kruskal–Wallis. Significant differences: *p < 0.05. **p < 0.01 are indicated in bold.

### 3.1 Qualitative findings

The relationships collected in the quantitative data were enriched from the emotionality provided by the qualitative data. The categories and their causal relationship with CS or CF are presented below.

#### 3.1.1 Difficulties in establishing a helping relationship with the user and CF

Recognition of a lack of a relational climate for support, especially with those who are not well disposed towards it, ends up causing them to question whether or not they should continue volunteering with that person so as not to end up feeling obliged to establish that relationship with the other “(…) the issue is whether you have a feeling with that person, and that person has a feeling with you” (1_FG1). Or, for example, as explained by volunteer 5_FG1, “Others have no one. Anyway. Their daily life is very monotonous, routine. And the truth is, loneliness is sometimes very complicated. So, these people create a bubble inside them, and it's tough to get inside that person so they can talk to you (…)”.

#### 3.1.2 Suppression of own emotions and CF

In the words of participant 4_FG1, “But the moment I'm with the person I have an emotion, which is there, that I try not to be moved or cry or anything, but when I leave, I say, I was moved. (…)”. Samples of situations emerged in the discourse where volunteers suppressed their negative emotions as if they denied them in order not to seem vulnerable, generating a case of emotional overflow due to their lack of management, “But, well, I already know in what project I am participating. And knowing their stories don't affect me at all, not at all.” (2_FG1). Likewise, they are also on the receiving end of the users' emotional offloading, acknowledging the strong impact this generates in them, “In front of their families, they don't cry, but in front of me, they do, and I tell them that sometimes crying is good (…)” (6_FG1).

#### 3.1.3 Self‐care and CS and CF

In both groups, behaviours related to self‐care and regulation of the emotional state were collected. It is significant that FG_1, whose participants presented a medium level of CF, were the ones who most manifested the need to be aware of their emotional state concerning volunteering. Participants shared, “That's why attention to emotions is so important to being a volunteer” (5_FG1); “I always say, emotion is fundamental. But don't let it drive you” (4_FG1). Yet it was the participants in FG_2 who widely recognised the phenomenon of CF and contributed coping strategies for it, “I give what I can right now, but I'm aware that the problem isn't solved” (9_FG2); “I think it's inevitable that you sometimes get worn down. There may, sometimes, be a connection, something from our past that touches us personally, but that sometimes makes it harder. Sometimes you must work on yourself emotionally. You see yourself in the other person because you see yourself, your own story (…) realize that when it's…. I experiencing an emotional toll, knowing how to manage it” (12_FG2).

Likewise, both groups recognised the exercise of volunteering in itself as a strategy of self‐care: “(…) And a lot of satisfaction, gratification, and emotional well‐being. It gives me a lot of good things” (10_FG2).

#### 3.1.4 Engagement and CS

Despite being aware of the high emotional load of their volunteering, they decide to continue despite the challenging experiences encountered therein. An example of this is the story of a volunteer who supported people in a drug dependence program, “When I saw his relapse, I was troubled for half an hour. But then I thought, either I behave like a volunteer or fall apart. And in half an hour, I changed completely; I thought, no if he's decided to give up, it's not my responsibility” (2_FG1). They recognise the positive feelings generated by acknowledging that they are committed to their volunteering, “For me, volunteering is an important part of my life and the fact of having the feeling that I'm on solid ground” (11_FG2); “(…) And then I feel a commitment. I commit to myself and the palliative care project” (12_FG2).

#### 3.1.5 Understanding function and CS

There are primarily stories linked to learning life lessons that provide satisfaction, “I've had a great learning experience, and all these people have given me wisdom. Generally, the sick people themselves. (…) And we've had abusers as users… all sorts, and when you see them, they're no more than just a person, and we aren't judges, and we can't judge. (…) And the wisdom they give you, they all teach you something… it satisfies you” (6_FG1). Learning that enables exploratory knowledge about the very meaning of life itself, “Learning the meaning of life through the contact this volunteering affords me. When people are near death, they've never been so alive in their life. Because they're aware they're alive and that we're here and we let each day go by” (12_FG2).

#### 3.1.6 Enhancement function, the feeling of giving back and CS

Both groups recognised the satisfaction generated by participating as volunteers. Therefore, their improvement in their state of mind, as well as in their perception of themselves, “For me, the experiences I've had have helped me be a better person” (12_FG2); “me too, also, living the volunteer experience has enabled me to feel that I'm a better person, that I can help” (13_FG2). It is worth noting the cases in which volunteers have previously been on the receiving end of the services offered by the organisation. In these cases, a positive feeling of giving back what was received at the time is generated “, and yes, it changed my life so much. And yes, I feel indebted. I feel indebted to this organization. My volunteer is a way of giving back and helping. It happened to me in the past” (3_FG1). However, self‐care should be taken into account in these cases as the line for arousing the feeling of CF is very fine.

#### 3.1.7 Protective function and CS

Stories emerge where awareness is raised of their reality and that of others, surrounded by pain or emotional discomfort, putting their problems into perspective: “[volunteering has helped me] to relativize everything. And to realize what is important” (2_FG1); “I've learned to minimize small problems. For example, not to complain about not sleeping because there is a leaking tap in the building. If I've had a person speaking with cancer (…), I see that man, and I think my concerns are trivial” (6_FG1).

#### 3.1.8 Social function and CS

Volunteering is recognised as an environment where positive relationships can be found. For instance, one volunteer finally embarked on a project, because one of her daughters initially took the step “(…) and when my daughter told me she was signing up for volunteering, I said I was going with her because I thought I felt like it. And I put it into practice” (10_FG2). Other individuals experience that it is an environment where new social relationships can arise, “(…) We [the volunteers] go on excursions together, (…) and talk about our experiences, about cases… And we've also formed incredible friendships” (12_FG2).

## 4 DISCUSSION

The sample follows the trend of volunteering in Spain (NGO Platform for Social Action in Spain, 2020), primarily women of advanced‐adult age (M = 58.33, SD = 16,868). It is possibly due to the improvement in the quality of life and life expectancy in Spain, which allows women to reach this time in better physical and cognitive conditions.

Our results show similarities with the work of Slocum‐Gori et al. (2013), regarding CF and CS levels. It seems that CS could be a reason for their continuity in the volunteer work, despite being in contact with pain, anguish or emotional discomfort. Despite this, the results obtained in this study are aligned with those presented by Di Marco et al. (2020), and no significant relationship was found between CS and CF. One explanation for this may be that all the participants scored high levels of CS, and therefore there was no dispersion of data that would allow a correlation, although a slight trend towards this was observed. Likewise, the qualitative data suggest a clear picture of these results since discussion group FG_1 argues that CS and CF can be felt simultaneously, with one feeling not affecting the other.

The negative relationship obtained between CF and Self‐Care could also be related to the high CS levels obtained. That is, the higher the level of Self‐Care, the lower the level of CF, which could generate compensation for positive feelings such as CS, as argued by Moreno‐Jiménez et al. (2013), Potash et al. (2015) and Pintado and Giannini (2016).

Volunteering is a non‐compulsory activity in opposition to professional practice would be. However, volunteering does involve a high level of engagement related to the increase in continuity over time, as also showed Garrosa et al. (2014), Kewes and Munsch (2019), Fourie et al. (2008), and Vecina et al. (2012). Likewise, the strong positive correlation between engagement and CS confirms the trend shown by Hidalgo (2018) or Meneghini et al. (2018). In the same line, results demonstrated that high levels of engagement with volunteering work offer effective retaining values like in Faletehan et al. (2020) and Mullan et al. (2021).

Regarding motivational functions, even though the values construct did not provide good reliability results, positive results were obtained for the other VFI constructs. With the motivation of Understanding showing the highest values and the fact that a large proportion of the sample had university studies, we are dealing with people interested in growth and learning, contributions that they recognise their volunteering offers them, therefore generating beneficial feelings like in Faletehan et al. (2020) or Ferreira et al. (2012, 2015). Likewise, as it is essentially a sample of retired people, as in Wilson and Musick (1997), the Enhancement function can be recognised to have a relevant role since, through their volunteering, they can feel involved and capable of contributing to society according to Russell et al. (2019), Jongenelis et al. (2020) and Wilding et al. (2021). Along the same lines, it is logical that the Career function should appear last since they have mostly finished their professional careers.

## 5 CONCLUSIONS

Being a volunteer in settings involving illness and suffering could imply a high emotional cost. However, the results obtained in this work show that the volunteers who spend time and support with hospitalised or lonely users, report highly positive feelings. CS, Engagement and the tree constructs comprising it are associated with social and health volunteering, as well as good levels of personal Self‐Care. Likewise, carrying out this type of volunteering fulfils all the functions of Understanding and Enhancement, generating even more positive feelings and adding a positive value to their experience of caring for others. These results are relevant to design organisational initiatives to motivate and retain volunteers in social and health programs. Thus, organisations should help volunteers to identify their positive and negative emotions, promoting self‐care practices and group interventions to share and work on those feelings. Finally, since high levels of CF may lead the cause to stop volunteering, social and NGO policies could encourage volunteering programs that prevent high CF.

### 5.1 Limitations

This study has some limitations. First, this study was developed prior to COVID‐19 pandemics. Thus, further studies should analyse the impact of lockdowns in the volunteering task, to know how volunteers and users experienced the interruption of their relationships.

Moreover, given the impossibility of having a precise census of the total population of volunteers in Mallorca, there is no data regarding the continuity of volunteers showing higher levels of CF. More studies related to the reasons why people decline to carry on volunteering may contribute to design initiatives to increase the retention of these individuals.

## AUTHORS' CONTRIBUTIONS

Conceptualisation, Cañas‐Lerma, Campos‐Vidal and Verger; methodology, Cañas‐Lerma and Verger; formal analysis, Cañas‐Lerma and Campos‐Vidal; research, Cañas‐Lerma, Campos‐Vidal and Verger. All authors have contributed to this manuscript.

## FUNDING INFORMATION

The authors received no financial support for the research, authorship, and/or publication of this article.

## CONFLICT OF INTEREST

The authors declared no potential conflicts of interest with respect to the research, authorship, and/or publication of this article.

## Data Availability

Quantitative Data Availability Statement: The quantitative data and syntaxis that produce the findings reported in this article are available at [https://osf.io/63c84/?view_only=cae13f3390c64ae896dd1457a0b06453]. Qualitative data that support the findings of this study are available from the corresponding author upon reasonable request.
